# Hsp27 and axonal growth in adult sensory neurons *in vitro*

**DOI:** 10.1186/1471-2202-6-24

**Published:** 2005-04-08

**Authors:** Kristy L Williams, Masuma Rahimtula, Karen M Mearow

**Affiliations:** 1 Division of Basic Medical Sciences, Memorial University of Newfoundland, St. John's, NL, A1B 3V6, Canada

## Abstract

**Background:**

Neurite growth can be elicited by growth factors and interactions with extracellular matrix molecules like laminin. Among the targets of the signalling pathways activated by these stimuli are cytoskeletal elements, such as actin, tubulin and neurofilaments. The cytoskeleton can also be modulated by other proteins, such as the small heat shock protein Hsp27. Hsp27 interacts with actin and tubulin in non-neuronal cells and while it has been suggested to play a role in the response of some neurons to injury, there have been no direct studies of its contribution to axonal regeneration.

**Results:**

We have investigated neurite initiation and process extension using cultures of adult dorsal root ganglion (DRG) sensory neurons and a laminin stimulation paradigm. Employing confocal microscopy and biochemical analyses we have examined localization of Hsp27 at early and later stages of neurite growth. Our results show that Hsp27 is colocalized with actin and tubulin in lamellopodia, filopodia, focal contacts and mature neurites and growth cones. Disruption of the actin cytoskeleton with cytochalasin D results in aberrant neurite initiation and extension, effects which may be attributable to alterations in actin polymerization states. Inhibition of Hsp27 phosphorylation in our cultures results in an atypical growth pattern that may be attributable to an effect of pHsp27 on the stability of the actin cytoskeleton.

**Conclusion:**

We observed colocalization of the phosphorylated and non-phosphorylated forms of Hsp27 with actin and tubulin in both very early and later stages of neurite growth from cultured adult DRG neurons. The colocalization of Hsp27 and pHsp27 with actin in lamellopodia and focal contacts at early stages of neurite growth, and in processes, branch points and growth cones at later stages, suggests that Hsp27 may play a role in neuritogenesis and subsequent neurite extension, and potentially in the patterning of this growth. Hsp27 has been reported to play a key role in modulating actin cytoskeletal dynamics as an actin-capping protein in non-neuronal cells. Our results suggest that this may also be the case in neurons and support a role for Hsp27 in neurite outgrowth via its phosphorylation state-dependent interactions with actin.

## Background

We know that various factors can influence and promote regeneration of peripheral axons. In addition to soluble factors (neurotrophins, cytokines and other growth factors), the extracellular environment in which growth occurs is critically important. Axonal regeneration does not occur to any great extent in the CNS, and while this is due to a number of factors, the most prominent is a non-permissive growth environment as well as an unavailability of appropriate growth-promoting factors. In the PNS, on the other hand, peripheral axons (both motor and sensory) generally regenerate quite well.

Growth factors and extracellular matrix (ECM) molecules like laminin act through cell surface receptors that activate often convergent signalling pathways to elicit neurite growth in sensory neurons [1]. Among the targets of these pathways are the cytoskeletal elements responsible for initiating and maintaining the structure of growing processes. Actin, tubulin and intermediate filaments all play a part in growth processes [2-4]. There are also a variety of other molecules that interact with these components to modulate or protect the cytoskeleton from deleterious stresses.

One class of molecules known to act as chaperones include the small heat shock protein family, of which heat shock protein 27 is a member. Hsp27, in addition to its roles in regulating apoptosis and protein folding, interacts with different cytoskeletal elements [5-9]. Much of this work has been carried out using non-neural cells, particularly fibroblast and epithelial derived cells. Part of its protective role in stressed cells has been attributed to its actions as an actin-capping protein [10,11]. Hsp27 has been reported to be a component of focal contacts, play an important role in smooth muscle contraction and be important for cellular migration in endothelial cells (reviewed in [12]). Rodent Hsp27 can be phosphorylated on 2 sites, Ser15 and Ser 86, although human Hsp27 has 3 serine phosphorylation sites (S15, S78 and S82) [13,14]. MAPKAP-K2, via its activation by p38 MAPK, is reported to be the Hsp27 kinase, although there are recent reports that PKC α,δ and cAMP-dependent kinase can also phosphorylate Hsp27 [15,16]. In terms of its influence on actin, pHsp27 acts to promote actin polymerization and stress fibre formation. It also has a role in protecting or stabilizing the actin cytoskeleton, although this appears to depend upon the nature of the pHsp [6,8,10]. Monomeric and non phospho-Hsp27 inhibit actin polymerization in vitro, while phosphorylated monomers and non-phosphorylated multimers have no effect on actin polymerization [10].

Prior reports and our own observations have suggested a role for Hsp27 in axonal growth or regeneration, in addition to its role in promoting neuronal survival. Hsp27 is upregulated after injury in DRG neurons in vivo and after dissociation in vitro ([17]; Dodge and Mearow, unpublished observations). Other injury models have shown increases in Hsp27 in Schwann cells and white matter columns [18] and it has been speculated that Hsp27 might be important in the neuronal response to injury and regeneration [17,19]. Of direct relevance to a potential role of Hsp27 in axonal growth are the recent reports indicating that Hsp27 and the related Hsp22 gene deletions are responsible for familial peripheral axonopathies [20,21].

In vitro models have been widely used to study the growth behaviour of neurite initiation and extension in both CSN and peripheral neurons. In many models, neurotrophin stimulation is required for neurite growth, although in most of these models neurotrophins are also required for survival. Another widely used paradigm involves the stimulation of plated neurons with soluble laminin or extracellular matrix preparations (eg., Matrigel^tm^), both of which elicit neurite initiation [22-24]. This approach is particularly useful in mature DRG neurons, where not all cells will respond to a given neurotrophin (for example, NGF). Regardless of how process formation is evoked, there appear to be several general stages that can be identified including the formation of lamellopodia, filopodia, and the eventual emergence of immature neurites with growth cones [2,4]. The cellular mechanisms responsible for these behaviours are not fully elucidated.

In our cultures of adult DRG neurons we have observed robust expression and distribution of Hsp27 in dissociated DRG neurons, particularly in neuritic networks and growth cones. These observations, along with the reported role of Hsp27 in modulating the actin cytoskeleton in other cells types, led us to investigate the potential role of Hsp27 in interacting with cytoskeletal elements in different stages of neurite initiation and extension. Our hypothesis was that Hsp27 associates with the cytoskeleton in neurons and plays a key role in regulating or fine-tuning the observed ability of the cells to initiate and extend processes in response to the appropriate stimuli.

## Results

### Laminin induces several identifiable stages of neurite initiation and growth

In order to investigate stages of neurite initiation and subsequent growth, we employed a laminin stimulation paradigm. As these neurons are adult, they do not require any added trophic factors for their survival, and therefore neither NGF nor any other neurotrophin was required to initiate growth in these experiments. Similar stimulation experiments using laminin or matrigel have been carried out using sympathetic neurons [22].

**Figure 1:**
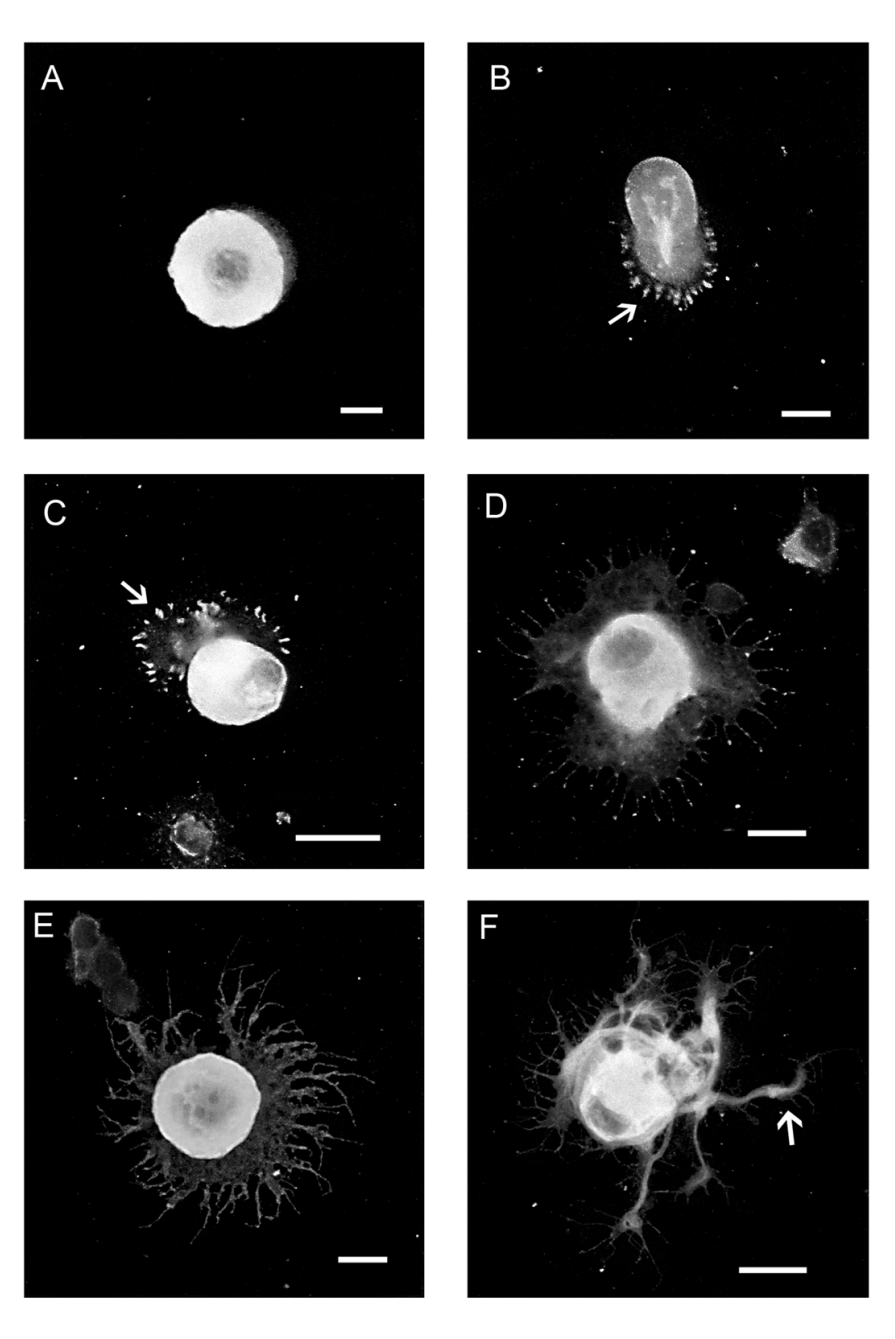
**Laminin stimulation elicits lamellopodia and process formation in adult sensory neurons. **DRG neurons plated on polylysine were stimulated with laminin in solution for 5 min, 15 min, 30 min, 1 hr, 6 hrs, 24 hrs. After fixation, neurons were stained with rhodamine-phalloidin to detect actin and images obtained using confocal microscopy. Panels A-F provide representative examples of the various stages of lamellopodia formation and eventual process protrusion, and show various distinctive stages in neuronal membrane expansion and neurite growth. At the earliest stages, lamellopodia are formed (A- 5 min, B- 15 min, C- 30 min) with evidence of focal contacts (arrows) at the leading edge of the lamellopodia (B, C). In D (1 hr) and E (6 hrs) filopodia begin to protrude from the lamellopodium around the circumference of the neuron. Eventually, these processes appear to coalesce into one or more neurites that continue to extend (F- 24 hrs, arrow). Scale bar – 20 μm.

Neurons were dissociated and plated on poly-lysine coated 16-well slides and allowed to adhere overnight (approx 18 hrs). Subsequently, the plating medium was removed and 50 μl of medium containing soluble laminin (40 μg/ml) was added to the cells. Control wells consisted of mock stimulation (eg., removal and replacement of laminin-free medium). Slides were fixed at 5, 15, 30 min and 1, 6 and 24 hrs after stimulation and subsequently processed for detection of actin, tubulin and Hsp27. Various distinctive stages in neuronal membrane expansion and neurite growth were observed and are summarized in Figure 1. One of the first steps is the appearance of a membranous expansion either around the whole soma or only from a particular portion of the cell body (A, 5 min). These lamellae are positively stained for actin (using phalloidin). Within 15–30 min, small sprouts extend from the lamellae and there are clear examples of focal contacts forming around the periphery of a lamellopodium (B, C, arrows). At later time points (1–6 hrs) some of the sprouts have elongated into filopodia and often have small growth cones associated with them (D, 1 hr; E, 6 hrs). Subsequently, neurites form and some are selected for extension by a process that is not well characterized (F, 24 hrs).

### Hsp27 colocalizes with actin and tubulin in the early stages of process initiation

**Figure 2:**
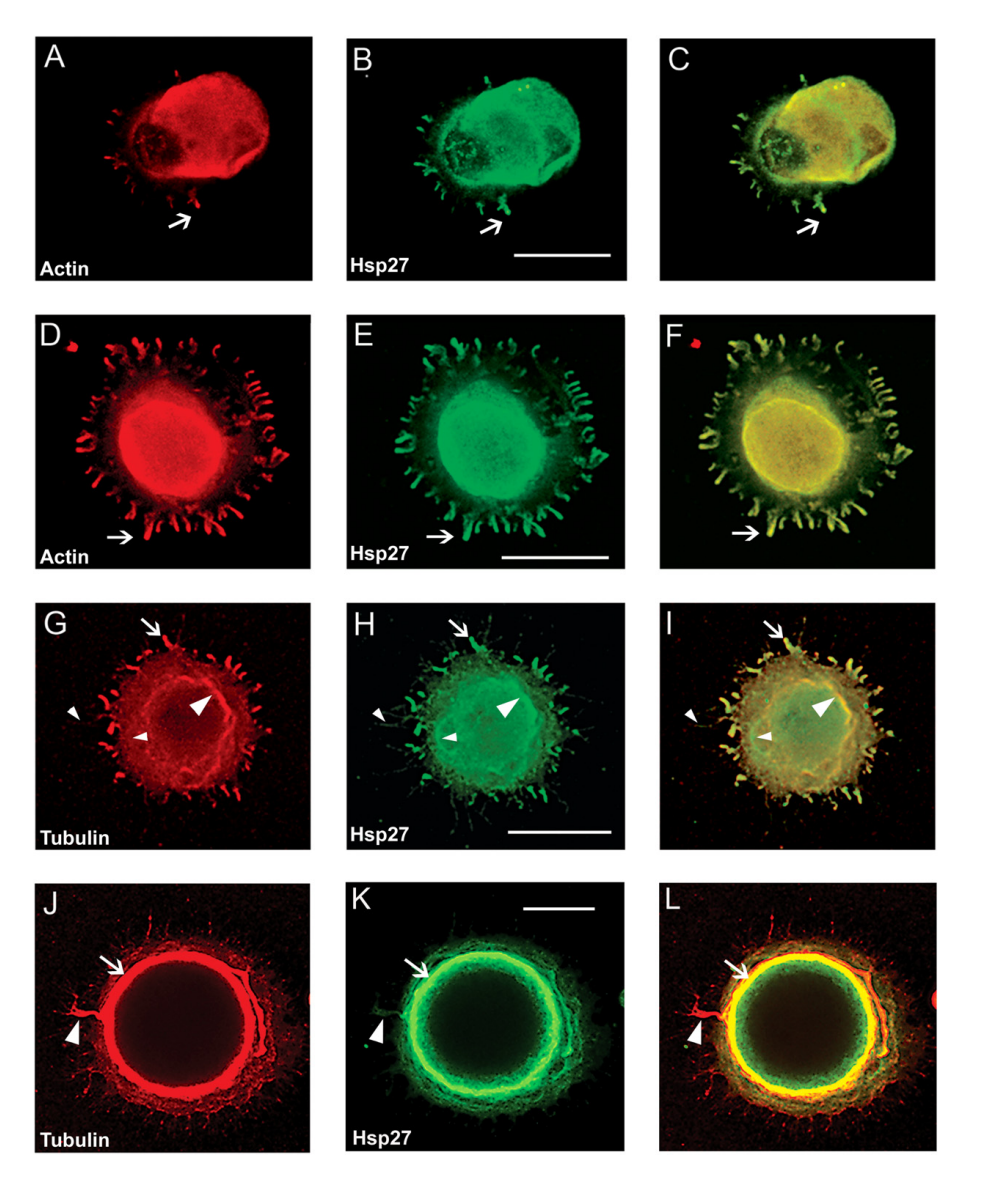
**Hsp27 co-localizes with actin and tubulin at early stages of neurite growth. **Neurons were plated on polylysine and stimulated with laminin for 1–6 hrs. Following fixation, neurons were labelled with rhodamine-phalloidin (red-A, D) or immunostained with antibodies directed against total tubulin (red – G, J) or Hsp27 (green -B, E, H, K). Images were obtained with confocal microscopy and panels C, F, I, L represent the merged images of the single channel images. Note colocalization of Hsp27 and actin in the lamellopodium (A-C, arrow) and in focal contacts observed in D-F (arrow). In panels G-I, there is some colocalization of the staining for tubulin and Hsp27 in the cortical region (large arrowhead) and in small processes emerging from the soma (arrow). In panels J-L, there is a more distinct colocalization of tubulin and Hsp27 in the cortical area (arrow, J-L) as well as in an obvious process that seems to be wrapping around the cell and finally extending (arrowhead, J-L). Scale bar – 20 μm

Based on our hypothesis that Hsp27 may play a role in process initiation or neurite growth, we examined the localization of Hsp27 in neurons in various stages of process formation using immunocytochemistry and confocal microscopy. Here, examples of the different stages as defined in the previous section (eg., lamellopodia, focal contacts, neurite emergence) were selected from cells stimulated with laminin for 1 or 6 hrs. In addition, because of the association of Hsp27 with actin and tubulin in non-neuronal cells [25-32], we also examined whether Hsp27 would colocalize with actin and/or tubulin in neurons. Representative results are presented in Figure 2. Panels 2A and D show actin (red) in contact points (arrows) located at the periphery of an lamellopodium at one end of the neuron in A and around the circumference of the lamellum of the neuron in D. Panels 2B and 2E show the corresponding images for Hsp27 (green). The merged images (2C, F) show that Hsp27 and actin appear to be colocalized in focal contacts.

In panels G-L, the cells were costained with antibodies for Hsp27 (green) and total tubulin (red). The neuron in Figure 2G and 2I is beginning to show progress from the lamellar stage toward the formation of small filopodia (G-I, arrow). Tubulin staining shows some concentration in the cortical area (G, large arrowhead). There is colocalization with Hsp27 in the cortical area (I, large arrowhead) and filopodia (I, arrow), although there are areas where there is little or no overlap with Hsp27 staining (G-I, small arrowhead). The neuron shown in Figure 2J and 2L displays a pattern that was seen consistently in several different experiments, with colocalization of tubulin and Hsp27 at the cortical area (arrow) and the emergence of a more discrete process (J-L, arrowhead).

### Phosphorylated Hsp27 is also localized with actin and tubulin at the early stages of process formation

**Figure 3:**
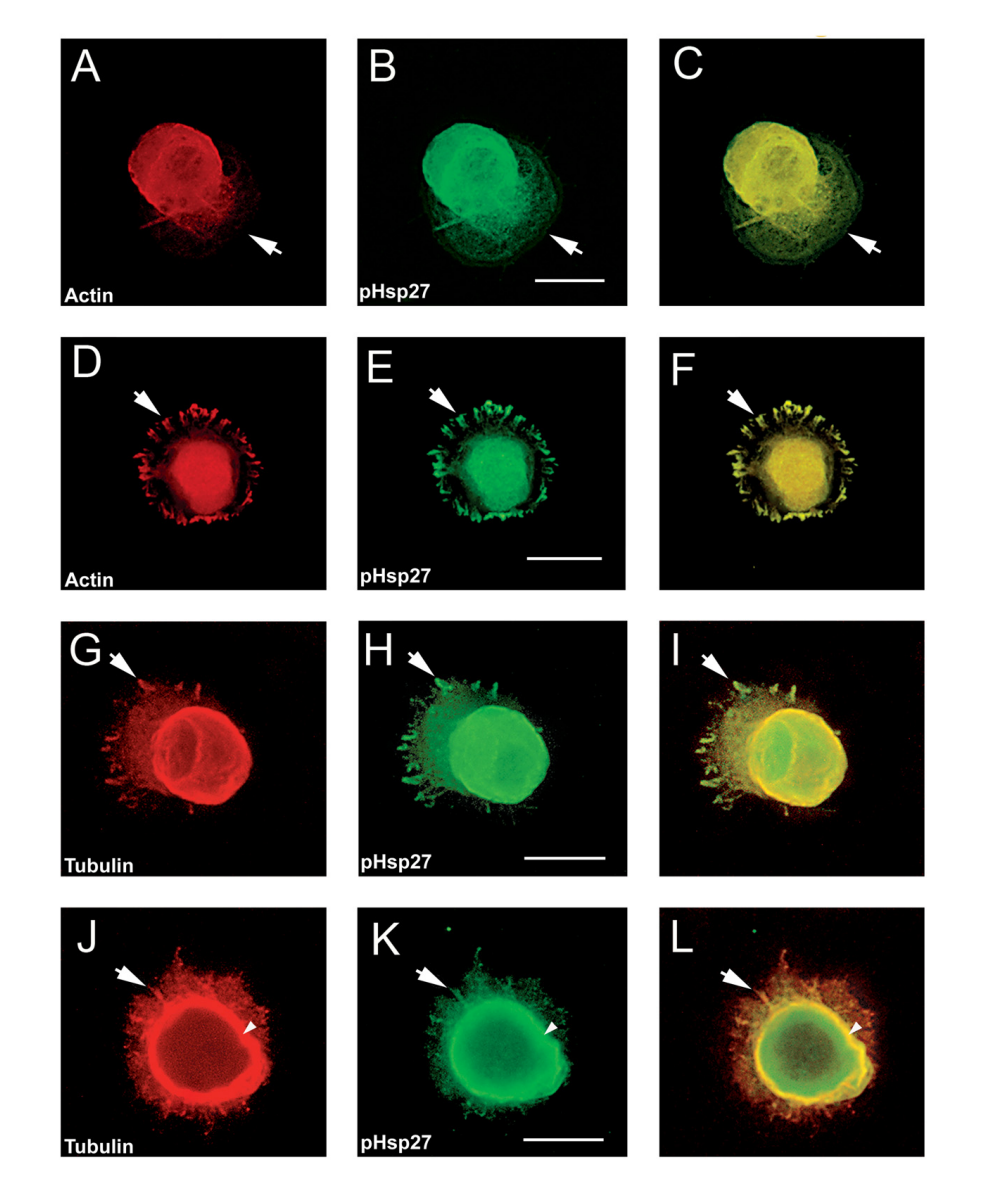
**pHsp27 also co-localizes with actin and tubulin at early stages of neurite growth. **Neurons plated on polylysine and stimulated with laminin for 1–6 hrs were also immunostained with antibodies directed against phosphorylated Hsp27 (pHsp27^Ser15^) to examine colocalization with actin or tubulin. A, D – rhodamine-phalloidin (red); B, E, H, K – pHsp27 (green); G, J – tubulin (red). The respective merged images are presented in panels C, F, I, L. Actin and pHsp27 appear to be colocalized in the body of the lamellopodium in A-C, but actin seems to be excluded from the leading edge (arrows). There is also localization of pHsp27 and actin in focal contacts (D-F, arrows). pHsp27 also colocalizes with tubulin in focal contacts (G-I, arrow), in a cortical ring (arrowhead) and in processes emerging from the cell body (J-L, arrow). Scale bar – 20 μm.

Hsp27 can be phosphorylated on 2 sites of rat Hsp27 (ser15 and ser 86), and this phosphorylation is reported to be important in the role of Hsp27 in its interactions with actin [31]. Using an antibody that recognizes Hsp27 phosphorylated on the ser15 site (pHsp27 ^S15^, ABR), we costained neurons at early stages (as defined above) of process formation for pHsp27 and actin (Fig 3A and 3F) and pHsp27 and tubulin (Fig 3G and 3L). Actin (A, D) and pHsp27 (B, E) show overlap in the lamellopodium (arrow) and in focal contacts (arrow, D-F). However, this is not complete, as noted by the exclusion of the pHsp27 from the leading edge of the lamellopodium (arrow, B, C). Tubulin (Fig 3G, J) and pHsp27 (Fig 3H, K) also colocalize in focal contacts (arrow, G-I), emerging filopodia (arrow, J-L) and in the cortical area (small arrowhead, J-L).

### Colocalization of Hsp27 and cytoskeletal elements in neurites and growth cones at later stages of neurite growth and extension

Our initial observations indicated that the majority of adult DRG neurons in culture display robust expression of Hsp27, not only in the cell bodies but throughout the neurites when present. Hsp27 expression in sensory neuron cell bodies as well as dendritic and axonal networks has been previously reported for in vivo expression [17,19,33]. In the present study, we further examined this distribution, particularly in terms of co-expression with actin and tubulin. Rather than using the soluble laminin stimulation paradigm employed in the experiments examining early events, in these experiments we plated the neurons directly onto laminin-coated slides and then fixed the cultures 24 hrs after plating. We have previously reported that when adult DRG neurons are cultured on surfaces coated with diluted growth factor-free Matrigel, or laminin, a relatively high percentage of the neurons display significant amount of neurite outgrowth by 24 hrs after plating [34].

**Figure 4:**
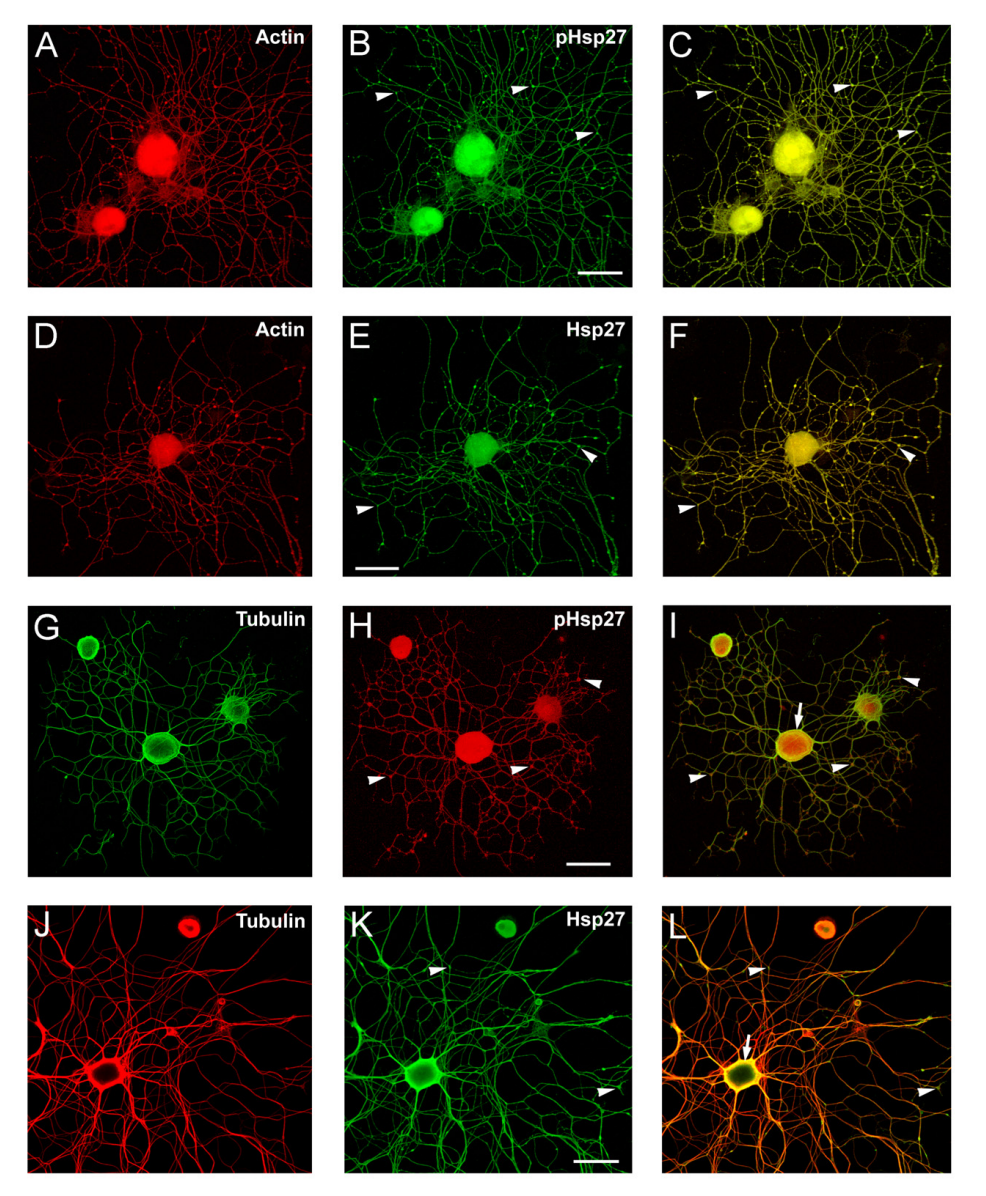
**Hsp27 continues to be expressed and localized with cytoskeletal elements in neurons and neuritic networks. **In these experiments, neurons were plated on laminin (no added neurotrophins) and fixed 24 hrs after plating. As shown in the images, many neurons exhibit extensive neuritic growth under these conditions. A-F: Neurons were labelled with rhodamine-phalloidin (A, D, red), and immunostained for pHsp27 (B, green) and Hsp27 (E, green); C, F – merged images. G-L: Neurons were immunostained for tubulin (G, green; J, red), pHsp27 (H, red), Hsp27 (K, green); I, L – merged images. Hsp27 and pHsp27 are expressed throughout the neuritic network, and there is colocalization of these with actin (C, F) and less so with tubulin (I, L). Note the accumulation of pHsp27 and Hsp27 at point of branching of neurites (arrowheads- B, C, E, F, H, I, K, L). The cortical colocalization of tubulin with pHsp27 and Hsp27 is still evident at this stage of neurite growth (arrows – I, L). Scale bar – 50 μm.

Figure 4A, F show representative neurons stained for actin (A, D) and pHsp27 (B) or Hsp27 (E) with the merged images displaying colocalization (C, F). The bottom panels show staining for total tubulin (G, J), pHsp27 (H) and Hsp27 (K) and the corresponding merged images in (I, L). As can be seen from the figures, Hsp27 is expressed throughout the neurons and associated neurites. As with the early stages of growth, tubulin strongly stains the cortical aspect of the cell soma as well as being present through out the processes. One interesting feature of the Hsp27 localization is the presence or local accumulation of Hsp27 and pHsp27 along with actin (but apparently not with tubulin) in branch or nodal points (arrowheads), suggesting a potential role in the pattern of neurite growth and branching. A previous publication reports beading of Hsp27 staining in dendrites of motor neurons and sensory neurons in sectioned material, although there was little discussion of the significance of this staining, other than to indicate that it was not associated with degenerating fibres [33].

**Figure 5:**
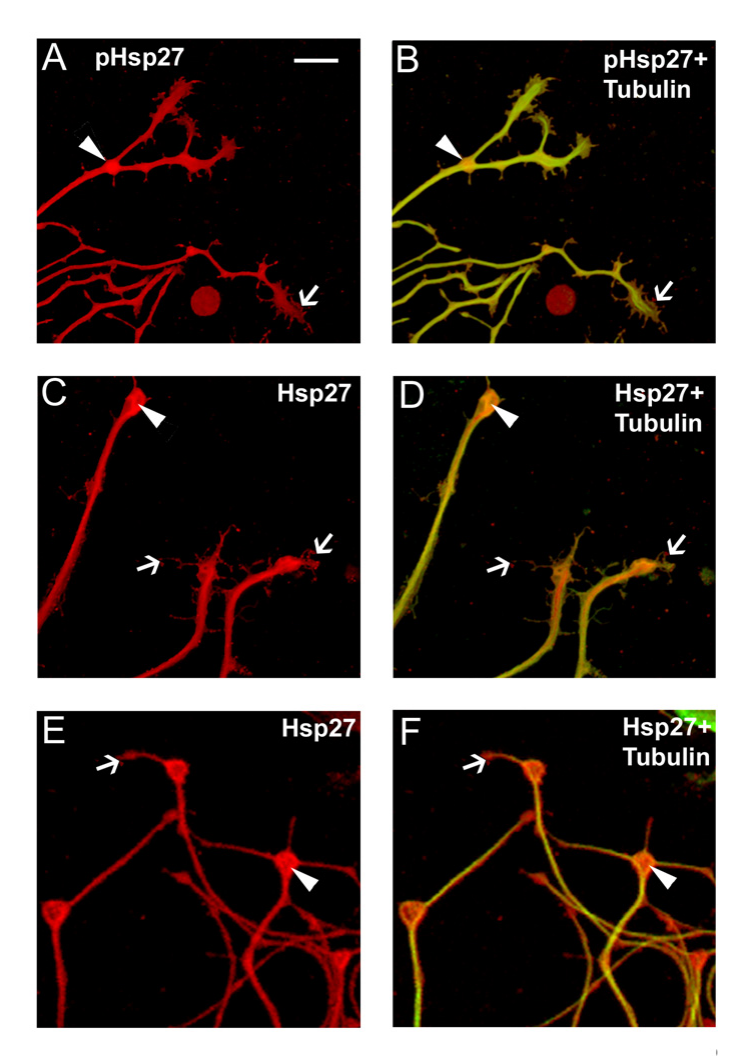
**Co-localization of Hsp27 and actin in growth cones of growing neurites. **Growth cones from neurons plated on laminin as outlined for Figure 4 were observed to express both pHsp27 and Hsp27. pHsp27 (A) and Hsp27 (C, E, red), shown together with tubulin (green-yellow) in the merged images (B, D, F), are present in growth cones and filopodia extending from the growth cones (arrows). There is also an accumulation in the core of growth cones and at points of neurite branching (arrowheads- A-F). Note that the tubulin staining does not completely overlap with pHsp27 or Hsp27, particularly in some of the extending filopodia (B, arrow) and the core of the growth cones in D, F (arrowheads). Scale bar – 10 mm.

We also noted that the Hsp27 and pHsp27 were strongly expressed in growth cones, further supporting an important role for Hsp27 not only in neurite initiation but also continued neurite extension. Figure 5 presents typical growth cones seen in the cultures described above. Different types of growth cones were observed with pHsp27 (A, B) and Hsp27 (C-F) being present in the core (arrowheads) of more expanded growth cones as well as in the filopodia (arrows). The growth cones in Figure 5C, F resemble the branch points noted in Figure 4, with an accumulation of an Hsp27 core and filopodia showing both Hsp27 and tubulin (merged images in D and F; tubulin, green and Hsp27, red). While the significance of this localization is not entirely clear, it is possible that one role of Hsp27 is to stabilize the cytoskeleton at these points where branching may occur (see below).

### Disruption of actin cytoskeleton with cytochalasin D results in aberrant neurite growth

Hsp27 has been suggested to play a key role in modulating actin cytoskeletal dynamics by acting as an actin-capping protein. In order to understand the role of Hsp27 in neuritic growth we decided to first examine the effects of disrupting the actin cytoskeleton integrity using cytochalasin D (CytD). Neurons were plated on laminin-coated slides and CytD was added to the medium 3 hrs post-plating (2 μM final concentration). Cultures were fixed 24 hrs later and examined for changes in neurite growth patterns and expression of Hsp27 and actin or tubulin.

**Figure 6:**
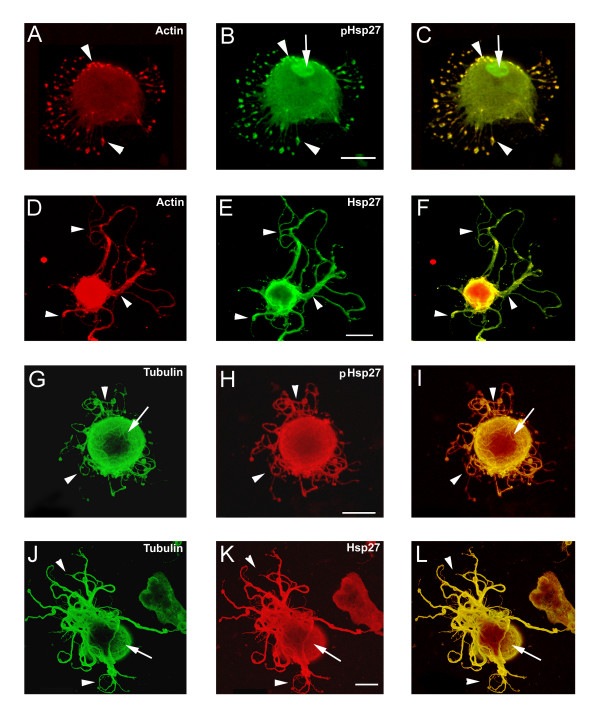
**Disruption of the actin cytoskeleton results in aberrant neurite growth. **Neurons plated on LN were treated with cytochalasin D (2 mM, added 3 hrs after plating), fixed 24 hrs later and stained for pHsp27 (B, H), Hsp27 (E, K), actin (A, D) or tubulin (G, J). The respective merged images are presented in panels C, F, I and L. The cytochalasin D treatment resulted in various atypical patterns of growth. One phenotype was the elaboration of numerous processes or microspikes as seen in panels A-C, with obvious accumulation of actin and pHsp27 especially at the tips of the microspikes (arrowheads); pHsp27, but not actin, accumulates in the nucleus (B, C, arrow). Abnormal process extension was also observed. In the neuron shown in D-F, some extension was observed although there was now less colocalization of actin with the Hsp27 (arrowheads). Panels G-L show tubulin staining along with either pHsp27 or Hsp27; the nature of the cytoskeletal network is clearer in these examples (arrows). Arrowheads point to atypical neurite growth, eg., lacking the usual radial branching pattern as seen in Fig 4. Scale bar – 20 mm.

Representative examples of the effects of CytD on neurons are presented in Figure 6. There was no discernible distinction between different sizes of neurons in their response to CytD; small, medium and large sized neurons displayed atypical process formation. Compared to the usual patterns of neuritic growth (Fig 4), neurons treated with CytD showed aberrant growth (Fig 6) including multiple processes emerging from the cell body (A-C), as well as stunted and disoriented neurites (D-L). In the cell displayed in Figure 6A, C, the processes show accumulation of actin (red) and pHsp27 (green) in their tips (arrowheads). Another example (D-F) shows several neurites that appear to have a disorganized internal structure resulting in the lack of the normal radial neurite extension and branching (arrowheads). In these examples, we used an antibody against actin, rather than phalloidin, in order to see total actin. In the bottom panels of Figure 6, two more examples are presented showing tubulin (green, G and J), pHsp27 (red, H) and Hsp27 (red, K) and the corresponding merged images (I, L). The cytoskeleton is more apparent in these latter examples, where the tubulin (and Hsp27) staining is clearly fibrillar in nature (arrows). Again, the disorganized and looping growth of neurites is apparent (arrowheads). In panels A-F, the actin antibody recognizes total actin, so even though CytD should disrupt the F-actin network, the antibody still detects G-actin.

### Inhibition of Hsp27 phosphorylation also results in aberrant neurite growth

Because of the reported role of Hsp27 phosphorylation in modulating the actin cytoskeleton, we wished to determine the effects of inhibiting p38 MAPK. p38 MAPK activity leads to the phosphorylation and activation of MAPKAP-K2, which acts as an Hsp27 kinase [35,36]. Inhibition of p38MAPK activity has been used to block phosphorylation of Hsp27 in the absence of direct inhibitors of MAPKAP-K2. We have thus used a combination of 2 commercially available p38 MAPK inhibitors (SB, SB203580 and SB202190, 10 μM) to investigate the potential contribution of phosphorylated Hsp27 to neurite growth.

**Figure 7:**
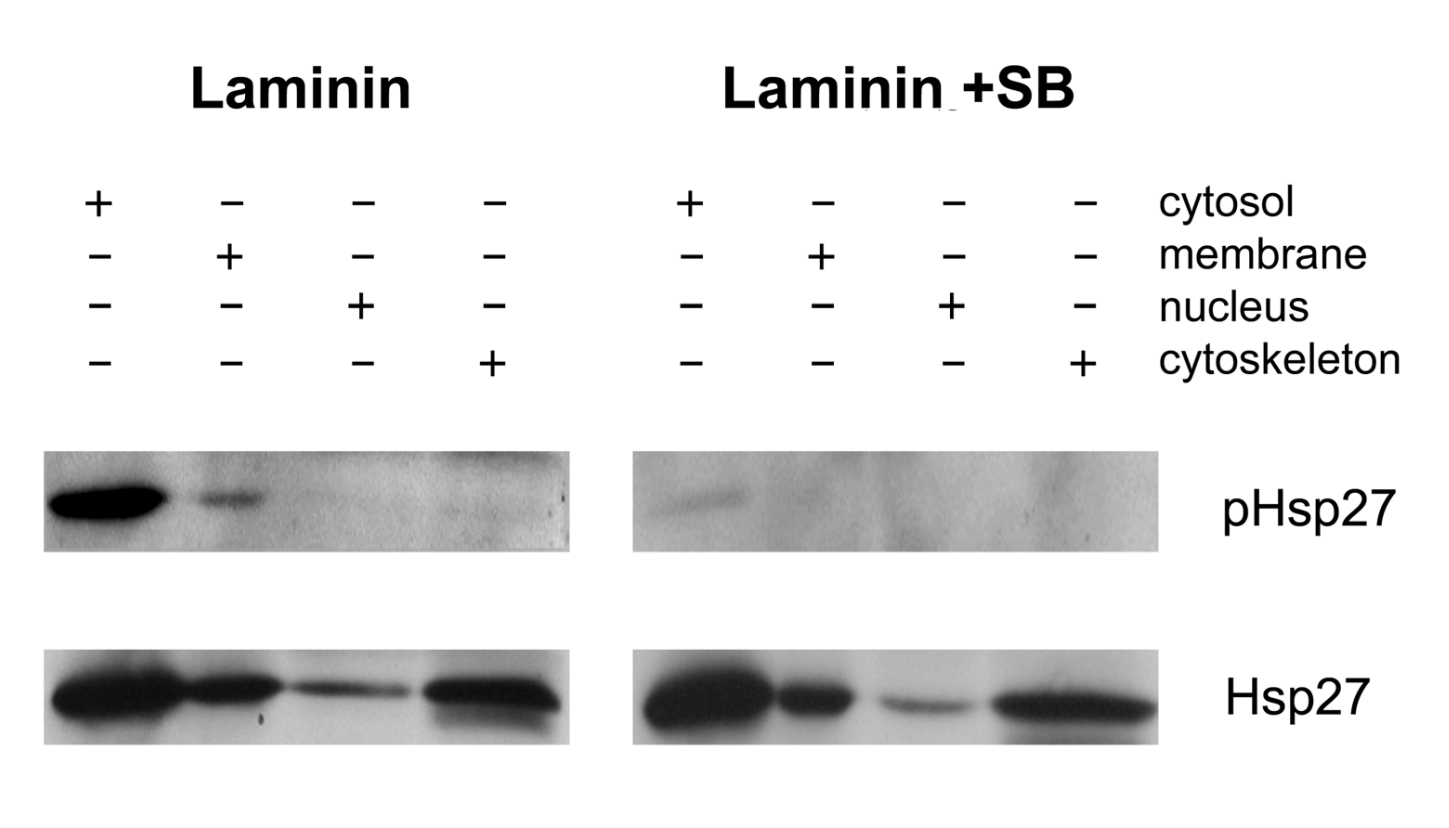
**p38 MAPK inhibition blocks phosphorylation of Hsp27. **Neurons plated on laminin were exposed to p38 MAPK inhibitors, SB203580 and SB202190 (10 mM each). Cells were sampled at 24 hrs post SB addition, using cellular subfractionation (as described in the Methods). The resulting protein from cytosol, membrane, nucleus and cytoskeleton fractions was electrophoresed and the blot subsequently probed for pHsp27 and Hsp27. Inhibition of p38 MAPK activity (laminin+SB) results in attenuation of the Hsp27 phosphorylation.

We initially determined whether the inhibitors were effective in preventing Hsp27 phosphorylation. Using larger scale cultures, neurons were plated on LN-coated 12-well plates and after 3 hrs the inhibitors were added; 24 hrs after SB addition, cell lysates were prepared as described in the Methods. For these experiments, we used a commercially available protocol to fractionate the cells into cytosolic, membrane, nuclear and cytoskeleton fractions. Following electrophoresis, the resulting blots were probed with pHsp27 ^S15 ^and total Hsp27 antibodies. The results of a representative experiment presented in Figure 7 show that inhibitors do indeed attenuate the phosphorylation of Hsp27. The blots also show that Hsp27 is found in the cytosolic, membrane and cytoskeletal fractions, while the pHsp27 is associated primarily with the soluble fraction.

Having determined that the inhibitors had the expected effects on pHsp27, we then plated the neurons on laminin-coated slides as for the previous experiments, and treated the cultures with SB 3 hrs after plating, fixed the cells 24 hrs later and carried out immunostaining for pHsp27, Hsp27, actin and tubulin as before.

**Figure 8:**
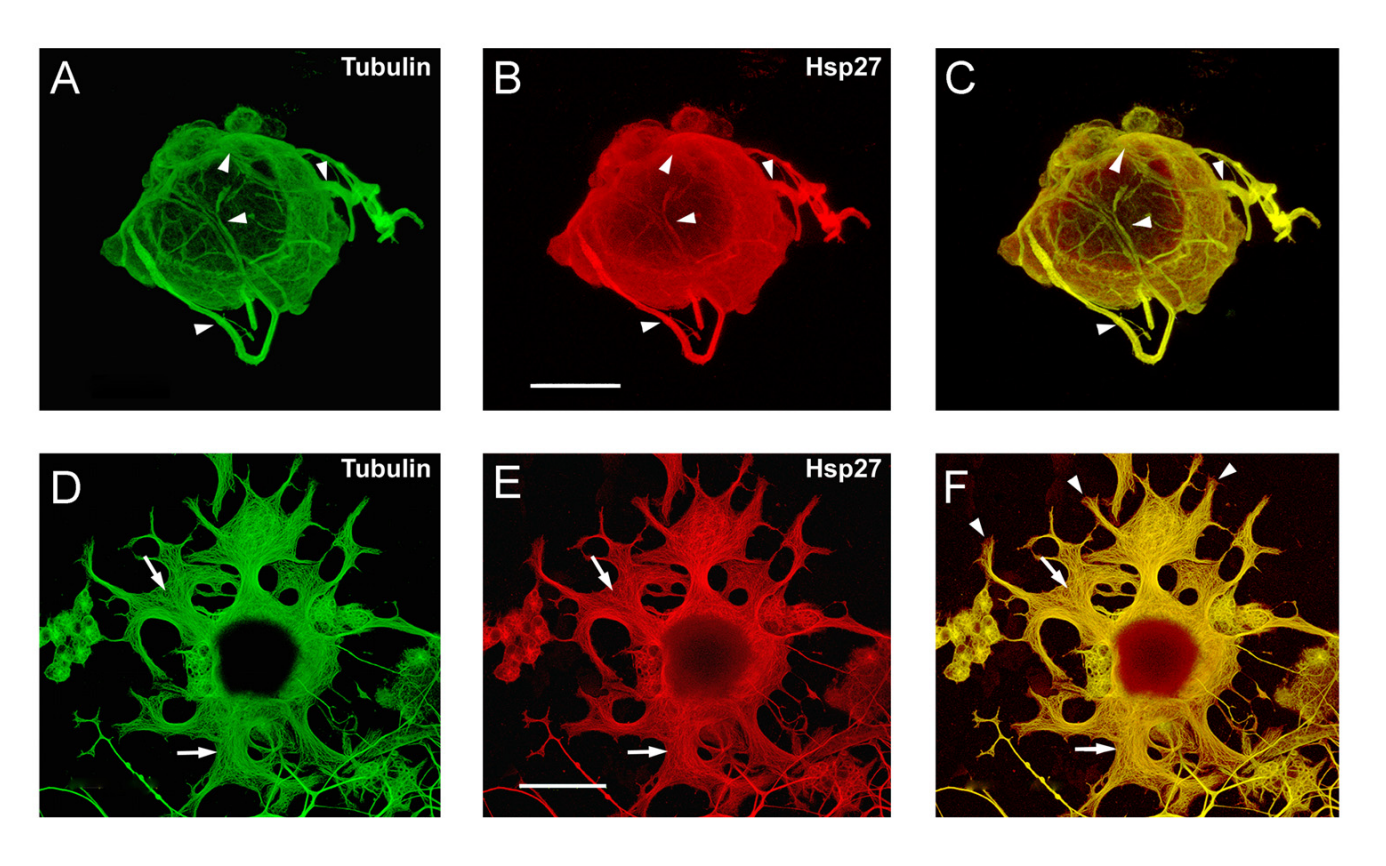
**Aberrant neurite growth following inhibition of Hsp27 phosphorylation. **Neurons plated on laminin were treated with p38 MAPK inhibitors (SB203580 and SB202190, 10 μM each, added 3 hrs after plating) and fixed 24 hrs later. Representative results are presented. Some neurons showed abortive extension, with neurites wrapping around the cell body, such as the example in panels A-C (arrowheads, A, tubulin, B, Hsp27, C, merged image). In another example, numerous processes were observed, but these terminated in large, flattened and splayed growth cones, as shown in panel D-F (D, tubulin, E, Hsp27, F, merged image). The fibrillar nature of the Hsp27 (E, arrows) and tubulin (D, arrows) is evident and the sites of colocalization with tubulin are also apparent (F, arrows). Also note that there is not a complete overlap of Hsp27 and tubulin at the tips of the growth cones (F, arrowheads). Scale bar – 20 μm.

Neurons treated with SB displayed clearly atypical neurite growth. The examples presented are representative of the various patterns of neurite growth observed. As with the CytD treatment, there was no discernible distinction between different sizes of neurons in their response to SB; small, medium and large sized neurons displayed aberrant process formation. In the neuron shown in Figure 7A, C, the neurites emerged from the cell body but wrapped around the soma (arrowheads) and appeared unable to undergo appropriate extension. Another common observation was the appearance of relatively short but flattened and expanded processes and growth cones. The example in Figure 8D, F is stained for tubulin (D, green) and Hsp27 (E, red), with the merged image (F) showing the disorganized nature of the cytoskeletal elements (arrows). In this example, note that tubulin does not have complete overlap with Hsp27 staining, particularly at the tips of the growth cones (F, arrowheads).

**Figure 9:**
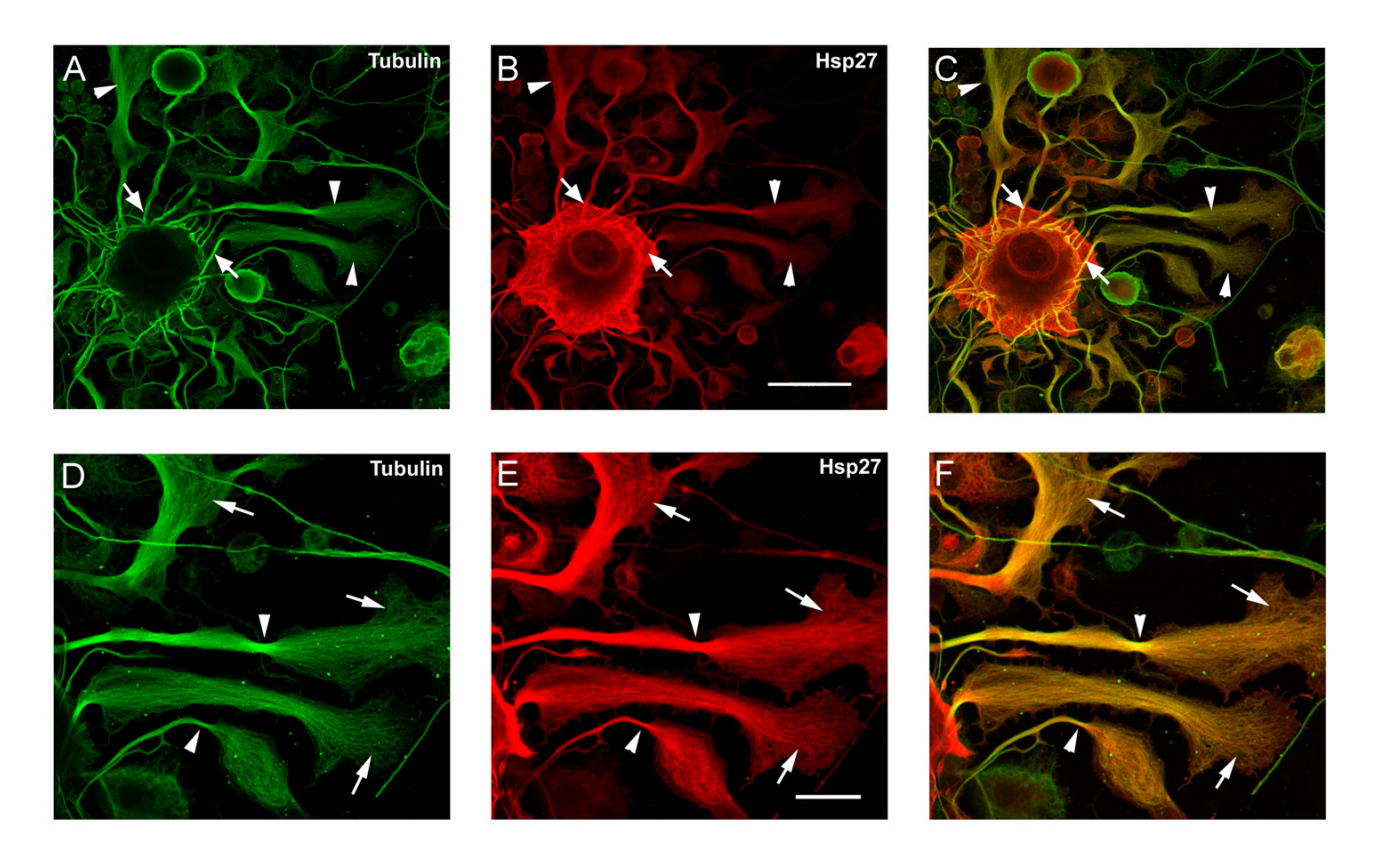
**Flattened growth cones and processes show co-localization of tubulin and Hsp27. **This figure shows another example of a neuron treated with the p38 MAPK inhibitors as outlined in Figure 8. Colocalization of Hsp27 (B, red) with tubulin (A, green) is apparent in the emerging processes (C, arrows), and in the flattened and splayed growth cones (A-C, arrowheads); scale bar – 50 μm. At a higher magnification (D-F), loss of microtubule bundling is observed (arrowheads) along with the fibrillar nature of Hsp27 and colocalization with tubulin (arrows); scale bar – 20 μm.

In addition, some neurons displayed extensive neurite growth, although this was again generally characterized by flattened and expanded processes and growth cones. Figure 9 presents such an example. This neuron has at least 7–8 processes extending from the cell body, all of which show process expansion. In panels A-C, Hsp27 (red) can clearly be observed colocalized with tubulin (green) in the processes emerging from the cell body (arrows). Areas of the fibrillar nature and overlap of Hsp27 and tubulin are also noted (arrowheads). In Figure 9D, F, larger magnification of the area generally noted by the arrowheads in A-C is shown. Here the splaying of the growth cones (arrows) and loss of cytoskeletal bundling is more apparent (arrowheads; compare processes observed in Figure 4 or 5 with those in Figure 9).

These results suggest that attenuation of the phosphorylation of Hsp27 can have adverse effects on the neuritic cytoskeleton, similar to those observed with Cyt D. Although our assumption (based on previous reports in the literature) is that the SB compounds block p38 MAPK activity, its downstream effects on MAPKAP-K2 and the subsequent inhibition of Hsp27 phosphorylation, it is possible that these compounds may have other inhibitory influences, or that they may be influencing the cytoskeletal elements through actions not involving Hsp27. While our data show that the SB compounds do inhibit phosphorylation of Hsp27, we cannot completely rule out effects on other signalling components, although at the concentrations we have used, the effects are reported to be specific for p38 MAPK inhibition, rather than any other additional kinases.

## Discussion

We describe early events in adult DRG neuron process formation in response to stimulation with the extracellular matrix protein laminin. Our data show that Hsp27 appears to associate with actin and tubulin in structures found at all stages of neurite initiation. Lamellopodia, filopodia, microspikes and focal contacts all displayed a colocalization of Hsp27 and actin or tubulin. The filamentous nature of the Hsp27 was quite clear in neurites and growth cones supporting the hypothesis that Hsp27 is associating with cytoskeletal elements.

Our results are similar to those described previously for neurite growth initiation and process extension in embryonic cultured CNS neurons. Culture studies of early neuritogenesis events in hippocampal neurons have provided information that demonstrates that events after initial cellular attachment to the substrate are quite similar among different cell types and indeed events in neurons are very similar to those in migratory fibroblasts [2,4,37]. The cells attach and are surrounded by a thin lamellopodium from which small extensions sprout. These extensions often have growth cones and display dynamic back and forth movements. At some point, one or more of these processes elongates, while the others remain stationary or retract. All the stages described by DaSilva and Dotti [2] and Dehmelt and Halpain [4] could be identified in our cultures of adult DRG neurons, suggesting that this process is intrinsic to all neurons.

Neurite protrusion requires the actin cytoskeleton, with lamellopodia being filled with an actin meshwork necessary for the appropriate adhesion and filopodia having actin bundles with the rapidly growing ends oriented towards the tips. Studies have shown that actin polymerizes at the leading edge of the lamellopodia, and then disassembles and recedes from the peripheral area [4,38]. This phenomenon influences growth cone advance and could likely play a role in neurite initiation as well. Microtubules may play a mechanical role in this since they invade the actin cytoskeleton in lamellopodia of various cell types. [2,4,38].

In cell-free assay systems, Hsp27 can act as an actin-capping protein which prevents the polymerization of actin and the assembly of F-actin [10,11]. Phosphorylation of Hsp27 leads to the loss of its ability to inhibit actin polymerization, and thus increases the rate and extent of actin polymerization and the formation of F-actin [6,8,10,11,39,40]. In addition to modulating the actin cytoskeleton, Hsp27 has also been reported to interact with both neurofilaments and microtubules in a phosphorylation-dependent manner [41,42]. Hsp27 has been inferred to stabilize not only actin, but also neurofilament and microtubules [31].

Phosphorylation of Hsp27 promotes the polymerization of actin and stress fibre formation [6,10,43]. Hsp27 is phosphorylated on 3 serines in the human Hsp27 (S15, S78, S82) and 2 in the rodent Hsp27 (S15 and S86 in mouse or S90 in hamster Hsp27). Hsp27 in unstressed cells exists as large oligomers, while upon phosphorylation Hsp27 dissociates in smaller species, including dimers and monomers [44,45]. In cell free assays, the unphosphorylated monomers of Hsp27 blocked actin polymerization, while the unphosphorylated oligomers and the phosphorylated monomeric form were ineffective. While the evidence based primarily on structural studies supports a role for phosphorylation of Hsp27 in stabilization of the actin cytoskeleton, a recent study has provided direct functional evidence that this is indeed the case [46].

Hsp27 phosphorylation is regulated by activity of the p38MAPK pathway, whereby p38 MAPK activation of MAP-kinase-activated protein-kinase 2/3 (MAPKAP-K2) leads to the phosphorylation of Hsp27 [36,44]. However, PKCδ and cGMP-dependent kinase have also been reported to phosphorylate Hsp27 in smooth muscle [47,48]. While the classical stress-activated signalling pathway activation of p38 MAPK regulates Hsp27 after heat-shock and other stresses, it is more likely that activation of p38 MAPK is downstream of the Cdc-42 and Rac activation of Pak1 with respect to neurite initiation and growth. For example, laminin can lead to p38 MAPK activation and Hsp27 phosphorylation, as previously reported for Schwann cells [49], and this is likely via Cdc-42 and Rac activation downstream of integrin-dependent signalling cascades [31]. Given the role of Rac and Rho in regulating actin dynamics in growth cones and the observations that inhibition of Rho promotes axonal growth on inhibitory substrates [50,51], the interactions of Hsp27 with Rho observed in smooth muscle cells [52-54] suggests an intriguing interplay among these components. Whether a similar interaction occurs in neurons or axons is not known.

Treatment of neurons with agents that disrupt the actin cytoskeleton result in aberrant neurite initiation and growth. Neurons treated with Cyt D, which caps existing actin filaments at barbed ends, consistently show rapid emergence of numerous neurites that elongate in a disoriented fashion [2,4,37]. Treatment of DRG neurons in our cultures resulted in similar aberrant growth, particularly at the early stages examined where numerous projections emerged from the neurons as early as 1 hr after laminin stimulation (data not shown); neurons examined at 24 hrs showed disoriented growth of processes (eg., Fig 6).

Since Cyt D acts to cap barbed actin filaments and non-phophorylated Hsp27 has been suggested to do the same, we reasoned that if pHsp27 was important for normal neurite initiation and extension, if we inhibited the phosphorylation of Hsp27 we might observe similar effects on neurite initiation. As shown in our results, attenuation of Hsp27 phosphorylation using the p38MAPK inhibitors, does indeed result in atypical growth patterns. At the early stages, results were similar to what we had observed with Cyt D (data not shown), and at later stages, neurite growth was again quite clearly aberrant (Fig 8, 9). Some neurons showed neurites that tended to wrap around the cell soma or extend in a disoriented fashion. Another consistent characteristic of the relatively short processes that did extend was the flattened and splayed nature of the neurites and growth cones. There appeared to be a lack of the appropriate actin and microtubular bundling that would result in normal neurite extension and growth cone dynamics (eg., compare Fig 4, 5 with Fig 6 and 8) [4,38]. We have inferred that effects of p38 MAPK inhibition on neurite growth were due to the inhibition of Hsp27 phosphorylation. A similar inhibition of neurite initiation by SB has been reported in PC12 cells [55]; interestingly, in this study induction of Hsp27 by heat shock promoted neuritogenesis. However, there may be effects on other cytoskeletal elements. Ackerley et al [56] have reported that p38 MAPK also phosphorylates neurofilaments in transfected COS cells, although they did not find any effect of p38 MAPK inhibition on neurofilament phosphorylation in cortical neurons.

There are relatively few reports of the interaction of Hsp27 with cytoskeletal elements other than actin. Hsp27 has been reported to associate with microtubules in HeLa cells [57] and in CHO cells [58]. In the latter report, overexpression of Hsp27 was shown to protect microtubules from heat shock and pH-induced collapse, although the contribution of pHsp27 to this effect was not reported [58].

pHsp27 also appears to be required for the migration of several cell types [29,59-61]. A recent study concluded that p38MAPK activation and Hsp27 phosphorylation played a key role in the regulation of actin polymerization, possibly by regulating the spatial organization of the lamellopodia by promoting branch formation at the leading edge and stability at the base [29]. They suggest that at the dynamic leading edge of lamellopodia, Hsp27 might promote branching by its actin-capping activity, while at the base p38MAPK remains active and Hsp27 is phosphorylated and might stabilize actin filaments.

Mutations of the small Hsp (Hsp22 and Hsp25/27) genes have been linked to axonal Charcot-Marie-Tooth disease and distal hereditary motor neuropathy (DHMN) [20,21]. This appears to be related to the disruption of the neurofilament networks by the aggregation of neurofilament proteins and collapse of neurofilament networks [20]. This study and recent commentaries [42,62] point to the importance of the small heat shock proteins like Hsp27 in regulating or modulating the function of cytoskeletal elements other than actin. However, the mechanisms underlying the function of Hsp27 and its regulation remain essentially unknown in neuronal cells.

Our results suggest that Hsp27 is necessary for the initiation of neurite outgrowth in DRG neurons. The data also suggest that phosphorylation of Hsp27 plays a key role in modulating the dynamic interactions of Hsp27 with cytoskeletal elements such as actin and tubulin to regulate the response of DRG neurons to environmental cues that mediate growth.

## Conclusion

Using immunocytochemistry, we observed colocalization of the phosphorylated and non-phosphorylated forms of Hsp27 with actin and tubulin in both very early and later stages of neurite growth from cultured adult DRG neurons. The colocalization of Hsp27 and pHsp27 with actin in lamellopodia and focal contacts at early neurite initiation stages, and in processes, branch points and growth cones in later stages suggests that Hsp27 may play a role in neurite initiation and extension and potentially in the patterning of this growth. While the mechanisms of action require further investigation, it is possible that one role of Hsp27 is to stabilize the cytoskeleton at potential sites of branching or sprouting. Hsp27 has been reported to play a key role in modulating actin cytoskeletal dynamics as an actin-capping protein in non-neuronal cells and our results suggest that this may also be the case in neurons. Neurons treated with cytochalasin D showed aberrant neurite growth patterns. Neurons treated with p38 MAPK inhibitors, which inhibit the downstream phosphorylation of Hsp27, also displayed either lack of neurite growth or failure of appropriate neurite extension. The similar results from the CytD and inhibition of Hsp27 phosphorylation support a role for Hsp27 in neurite outgrowth via its phosphorylation state-dependent interactions with actin.

## Methods

### Neuronal cultures

Dorsal root ganglia (DRG) from young adult (5–6 wk) Sprague-Dawley rats (Memorial University of Newfoundland Vivarium and Charles River Canada, Montreal, QC) were dissected and dissociated using modifications to techniques described previously [34,63]. Briefly, ganglia from all spinal levels were removed and the roots trimmed, and subsequently incubated in 0.25% collagenase for 45 min, followed by 0.25% trypsin for 20 min (Invitrogen/ Gibco BRL, Burlington, Ont). Dissociated neurons were suspended in serum-free Neurobasal medium (NB, Invitrogen) supplemented with 100 U penicillin/streptomycin, B27 supplement (Invitrogen), and 20 μM cytosine arabinoside (modified NB). This suspension was then layered on top of a 28% Percoll solution (Amersham Bioscience, Baie d'Urfe, QC) in 15 ml conical tubes, centrifuged at 400 g for 20 min at room temperature. Pellets were then carefully extracted with a sterile pasture pipette, placed in a fresh tube, washed with the previous suspension media and centrifuged to remove any remaining Percoll. Neurons were plated in Lab-Tek 16-well chamber slides (Nunc International, Naperville, NC) for neurite growth assessment or 12-well plates for Western blotting and incubated at 37°C, 95% O_2 _and 5% CO_2_. Slides and culture plates were coated with poly-lysine (PL, 1 μg/ml, BD Bioscience, Bedford, MA) or laminin, (LM, 20–40 μg/ml, Invitrogen) where appropriate. The neurons were cultured in modified serum-free NB alone with no added growth factors.

### Immunocytochemistry

Neurons were fixed in 4% paraformaldehyde (pH 7–7.4) in PBS for 20 minutes, permeabilized with 0.1% Triton-X-100 and blocked with 5% normal goat serum in PBS. Antibodies used were as follows: Hsp27 (SPA-801, Stressgen Corp, Victoria, BC) and phospho-Hsp27^S15 ^(PAI-018, Affinity BioReagents, Golden, CO), total tubulin (Sigma-Aldrich, St. Louis, MO), actin (Sigma-Aldrich). It should be noted that the Hsp27 antibody recognizes both the non-phosphorylated and phosphorylated Hsp27, while the pHsp27 antibody only recognizes the phosphorylated form. We have also tested two other pHsp27 antibodies (UBI and Santa Cruz, see [63]), but have found the Affinity Bioreagents Antibody to be better for immunostaining.

Cells were incubated with the primary antibodies at 4°C for 16–20 hrs, followed by Cy2 or Cy5-tagged secondary antibodies (Jackson Immunoresearch Labs, West Grove, PA). In some experiments, cells were also labelled with rhodamine-phalloidin after antibody incubation (Sigma-Aldrich). The slides were coverslipped with glycerol and imaged with confocal laser scanning microscopy using z-stage scanning and image stacking. Stacked digital images were imported into Adobe Photoshop for compilation into the final composite figures.

### Laminin stimulation and neurite growth initiation

Neurite initiation was assessed in two ways. The first series of experiments employed neurons plated on laminin-coated slides, with the cells being fixed and analyzed for outgrowth parameters (lamellopodia, filopodia and neurite initiation and extension) at 24 hrs after plating. In a second series of experiments, the neurons were first plated on polylysine coated slides and allowed to stabilize overnight prior to being stimulated with soluble laminin (20 μg/ml in basal medium). Following the addition of the laminin solution, cells were then fixed at 5, 15, 30 min, 1 hr, 6 hr, and 24 hr. After fixation, cells were immunostained and analyzed as described above.

### Inhibitor experiments

SB 203580 and SB 202190 (10 μM, Calbiochem/EMD Biosciences, San Diego, CA) were used to inhibit p38 MAPK activity, in order to assess the contribution of phosphorylated Hsp27. Inhibitors were added 1 hr prior to laminin stimulation. For the 24 hr cultures, the inhibitors were added 2–3 hrs after plating the cells on laminin-coated slides and retained in the medium for the extent of the experiment (usually 24 hrs). Cytochalasin D (2 μM, Sigma) was also used in longer term experiments, and was added 3 hrs after plating and maintained in the medium for the extent of the experiment (24 hrs)

### Immunoblotting

For Western analyses, neurons were plated in 12-well plates that had been coated with polylysine alone or with laminin, depending on which experimental paradigm was used (see above). Neurons were subsequently processed according to our established procedures [34,63]. Cellular fractionation was carried out using a subcellular protein extraction kit (ProteoExtract, Calbiochem/EMD Biosciences, San Diego, CA) to isolate cytoplasmic, membrane, nuclear and cytoskeletal fractions. This protocol involves sequential isolation of these fractions using specific buffer systems (proprietary, as supplied by the manufacturer) to lyse cells in situ in the tissue culture plates. Subsequently, protein concentrations were determined for the fractions using the BCA protein assay (Pierce Chemicals, Rockford, IL.). Equivalent amounts of protein (40 μg protein) were loaded in each lane. Following transfer to nitrocellulose, the blots were first stained with Ponceau Red to assess protein loading, and subsequently probed with the following antibodies: phospho-Hsp27^S15 ^(PAI-016, Affinity Bioreagents) and Hsp27 (SPA-801, Stressgen),. Blots were cut and reprobed sequentially, and visualized with ECL reagents (NEN, Boston, MA) and exposure to X-ray film (Cronex MRF Clear base, Agfa Corp, Greenville, SC). Developed films were subsequently digitized and densitometrically analyzed with a cyclone ChemiImager and AlphaEase software. Digital images of the blots were used to make composite figures with Adobe Photoshop graphics software (Adobe Corp, MountainView CA).

## Authors' contributions

KLW carried out neurite growth and confocal analyses, as well as the Western blotting experiments. MR provided technical assistance with aspects of the work, most notably preparing neuronal cultures. KMM wrote the manuscript and was responsible for the experimental concept, design and overall supervision of the experiments, as well as carrying out some of the confocal analyses.
